# PI3K inhibition potentiates Bcl-2-dependent apoptosis in renal carcinoma cells

**DOI:** 10.1111/jcmm.12019

**Published:** 2013-02-07

**Authors:** Shudong Zhu, Matthew B Cohen, Jeffrey D Bjorge, James W Mier, Daniel C Cho

**Affiliations:** a Division of Hematology and Oncology, Beth Israel Deaconess Medical Center, Harvard Medical SchoolBoston, MA, USA; b School of Biological Science and Technology, State Key Laboratory of Medical Genetics, Central South UniversityChangsha, China; c Department of Biochemistry and Molecular Biology, Southern Alberta Cancer Research InstituteCalgary, Canada

**Keywords:** PI3K, Bcl-2, apoptosis, renal carcinoma cell, XIAP

## Abstract

Inhibitors of PI3-K/Akt are currently being assessed clinically in patients with advanced RCC. Identification of therapeutic strategies that might enhance the efficacy of PI3-K/Akt inhibitors is therefore of great interest. As PI3-K inhibition would be expected to have many pro-apoptotic effects, we hypothesized that there may be unique synergy between PI3-K inhibitors and BH3-mimetics. Towards this end, we assessed the combination of the PI3K inhibitor LY 294002 and the Bcl-2 family inhibitor ABT-737 in RCC cell lines. We found that the combinatorial treatment with these agents led to a significant increase in PARP cleavage and cell death in all RCC cell lines. The synergized cell death was correlated with decreased levels of Mcl-1 and XIAP, and increased levels in Bim, and appears critically dependent upon the activation of caspase 3 and 8. The enhanced lethality observed with the combination also appears dependent upon the regulation of XIAP, Mcl-1 and Bim levels. Our results suggest that the combination of PI3-K inhibitors with BH3-mimetics may be a viable therapeutic strategy in RCC.

## Introduction

Inhibitors of phosphatidylinositol 3-kinase (PI3-K) are in active clinical development for the therapy of patients with metastatic renal cell carcinoma (RCC). This follows on the heels of the recent demonstration of clinical activity and subsequent FDA approval of two allosteric inhibitors of mammalian target of rapamycin (mTOR), a kinase regulated downstream of PI3-K, in patients with advanced RCC [1, 2]. However, while there is optimism for these novel agents in RCC, it remains likely that only a subset of patients will derive substantial clinical benefit and efforts must be focused on mechanisms of resistance as well as potential rationale combinations with other molecularly targeted agents.

Although therapeutic rationale for the assessment of PI3-K inhibitors in RCC is in part based on upon expectation of downstream suppression of mTOR, inhibition of PI3-K would be expected to have a much broader range of effects. PI3-K, primarily through its downstream effector Akt (Protein Kinase B), regulates the function of a broad array of proteins involved in cell growth, proliferation, motility, adhesion, neovascularization and cell death [3]. In particular, Akt affects apoptosis susceptibility by regulating the activity of both apoptotic proteins and transcription factors. Akt directly phosphorylates several pro-apoptotic proteins, including procaspase 9, the Bcl-2 family member BAD, and apoptosis signal regulating kinase-1 (ASK1), resulting in their inactivation [4–6]. Akt also phosphorylates and differentially regulates transcriptional factors controlling expression of apoptotic genes, negatively regulating factors promoting expression of death-associated genes [*e.g*. Forkhead (FOXO) family member] and positively regulating genes promoting survival (*e.g*. NF-κB) [7, 8]. Akt activation has also been shown to enhance expression of the anti-apoptotic gene *bcl-2* through phosphorylation of cyclic AMP response binding protein (CREB) [9]. Thus, the phosphorylation of several known substrates by Akt increases cellular resistance to programmed cell death.

Coincident with efforts to develop inhibitors of PI3-K/Akt have been efforts to develop BH3 mimetics which, like BH3-only family members, bind to and sequester anti-apoptotic Bcl-2 family members. Perhaps the best characterized of these agents is ABT-737 which functions primarily by binding to Bcl-2, Bcl-xL and Bcl-w. ABT-737 has been shown to induce apoptosis in several pre-clinical models, both as a single agent and in combination with chemotherapies and molecularly targeted agents [10]. Given the aforementioned dependence of many elements of intrinsic apoptotic pathway upon PI3-K/Akt activity, there may be unique synergy between inhibitors of PI3-K/Akt and BH3-mimetics such as ABT-737.

In this manuscript, we describe the additive effects of the ABT-737 and PI3-Kinase inhibition. We demonstrate that concurrent treatment of RCC cell lines *in vitro* with ABT-737 and the PI3-Kinase inhibitor LY 294002 results in dramatically increased cytotoxicity than observed with either agent alone. This additive lethality appears dependent upon the induction of BIM and concurrent downregulation of both XIAP and Mcl-1.

## Materials and methods

### Cell lines and reagents

Human RCC cell lines 786-O (VHL−/−, PTEN-null), 769-P (VHL−/−, PTEN wt) and Caki-1 (VHL and PTEN wt) were obtained from the American Type Culture Collection. The 769-P, 786-O and stable cell lines (786-O-X, for stable expression of XIAP; 786-O-M, for stable expression of Mcl-1; 786-O-XM, for stable expression of XIAP and Mcl-1) were cultured in RPMI 1640, and Caki-1 in McCoy's 5A. All media contained 10% foetal bovine serum (FBS), 4 mM glutamine and 50 μM gentamycin. 786-O cells were transfected with XIAP-pcDNA3 and selected with G418 to derive XIAP stably transfected cells (786-O-X). XIAP Gene expression (approximately twofold higher expression relative to parental wild-type control cells) was confirmed by Western blot analysis. 786-O cells were also cotransfected with Mcl-1-pBabe and an enhanced green fluorescent protein plasmid. Three clones with green fluorescence were examined by Western blot analysis to confirm Mcl-1 Gene expression (approximately twofold higher expression relative to control cells) and these three cell clones were mixed in equal numbers to generate the 786-O-M cells used in our experiments. 786-O-M cells were transfected with XIAP-pcDNA3 and then selected with G418 to derive the 786-O-XM cells, and XIAP and Mcl-1 expression were confirmed by Western blot (up to twofold higher expression relative to control cells). Cells were incubated at 37°C at 5% CO_2_. LY 294002 was purchased from Cell Signaling (Beverly, MA, USA) and ABT-737 was obtained through a Material Transfer Agreement with Abbott Pharmaceuticals. Both regents were dissolved in DMSO for *in vitro* assays.

### Western blot

Cells were treated as described in Results and then lysed in RIPA lysis buffer (Cell Signaling) supplemented with sodium fluoride (10 μM) and phenylmethylsulfonyl fluoride (100 μg/ml). Proteins were separated on SDS-PAGE gels and transferred to nitrocellulose membranes. The blots were probed with specific primary antibodies and secondary conjugates followed by incubation with SuperSignal substrates (Pierce, Rockford, IL, USA). Phospho and total Akt, NOXA, PARP, vinculin, Mcl-1, Bim, XIAP, caspase 3, 7, 8, 9, cytochrome C and phospho-GSK3 antibodies were purchased from Cell Signaling. CoxIV antibody was from Abcam.

### Caspase activity

Caspase activity was determined using a fluorometric caspase assay kit (Abcam, Cambridge, MA, USA) and expressed as fluorescence as measured at the emission wavelength of 505 nm [11].

### Cell death assay

The adherent cells were detached from cell culture dishes by treatment with trypsin and then combined with the non-adherent cells. Propidium iodide (5 ng/ml) was added to the cells, and after incubation of 30 min. at room temperature in the dark, the cells were analysed by flow cytometry with a BD Biosciences FACScan. The percentage of cells staining positive was recorded to represent the level of cell death induced in each experiment.

### BAX/BAK activation assay

Cells were treated with DMSO, LY 294002 and ABT-737 for 24 hrs. Flow cytometric analysis of BAX and BAK Activation were as described in Panaretakis *et al*. [12].

### Subcellular fractionation

Mitochondria were isolated from the cytosolic fraction of 786-O cells using the Mitochondria Isolation Kit for Mammalian Cells (Thermo Scientific, Rockford, IL, USA).

### Transfection

786-O cells were transfected with control siRNA or Bim siRNA (200 nM; Santa Cruz, Santa Cruz, CA, USA), using Lipofectamine 2000 (Life Technologies, Grand Island, NY, USA), as per the manufacturer's instructions. Mediums were changed after 6 hrs. Cells were treated with drugs 24 hrs later.

### Drug treatment

Cells were exposed to 50 μM of LY 294002 (or 0.1% DMSO) for 30 min. followed by addition of ABT-737 (or 0.1% DMSO) in concentrations as indicated in the results. Cells were then either lysed after 3 hrs of treatment and analysed by Western blot or collected after 18 hrs of treatment for cell death assays.

### SLP infection

shRNA lentiviral particles (SLP) from Biosystems targeting individual genes and shRNA gene silencing protocol from Santa Cruz were used.

### Statistical analysis

Statistical analysis was performed with EXCEL. Results for cell death assays were reported as mean ± SD. Correlations between sample groups were calculated by unpaired, one-tailed *t* test. Differences with *P* < 0.05 were considered significant.

## Results

### PI3K inhibitor LY 294002 and Bcl-2 family inhibitor ABT-737 synergize to induce cell death in RCC cells

**Fig. 1:**
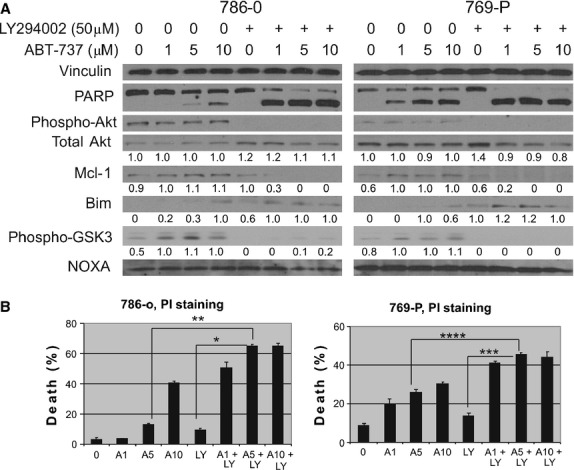
LY 294002 and ABT-737 synergize to induce cell death in RCC cells. 786-O and 769-P cells were first treated with 50 μM LY 294002 in 0.1% DMSO or control 0.1% DMSO for 30 min., followed by addition of ABT-737 of indicated concentration or control 0.1% DMSO for 3 hrs. Vinculin, PARP, Phospho-AKT, AKT, Bim, Mcl-1 NOXA and phospho-GSK3 levels were assessed by immunoblotting with respective antibodies. The experiment was performed three times with similar results and representative blots were shown (**A**). Apoptosis was also assessed by flow cytometry with PI nuclear staining after 18 hrs incubation, *N* = 3 (**B**). *,**,***,****P < 0.001, unpaired, one-tailed *t* test.

To assess the *in vitro* effects of LY 294002 and ABT-737 on intracellular signalling and cell viability, RCC cell lines 786-O and 769-P were exposed to increasing concentrations ABT-737 in the presence and absence of LY 294002 at a dose of 50 μM. The dose of LY 294002 was selected as this was the dose required to completely suppress S6 phosphorylation in RCC cell lines (data not shown). As shown in Figure 1A, treatment of RCC cell lines with LY 294002 reduced the phosphorylation of AKT^Ser473^ and phosphorylation of AKT substrate GSK3, confirming that PI3K activity was suppressed. While the treatment of RCC cell lines with LY 294002 alone resulted in minimal induction of cell death, treatment with increasing concentrations of single-agent ABT-737 resulted in increasing cell death as determined by both PARP cleavage and PI staining (Fig. 1). Concurrent treatment of RCC cells with ABT-737 and LY 294002 resulted in significant additive lethality by both PARP cleavage and PI staining. This was most apparent when LY 294002 (9% apoptosis) were combined with ABT-737 at 1 μM (4% apoptosis) and 5 μM (13% apoptosis) to produce apoptosis of 50% and 65%, respectively, (Fig. 1B) in 786-O cells, indicating a synergy between LY 294002 and ABT-737 in causing cell death.

To investigate the mechanisms underlying the synergistic apoptotic effect, we have examined a panel of several apoptosis-related factors including NOXA, Mcl-1 and Bim. While NOXA levels were invariant during the treatment, a reduction in Mcl-1 levels and an induction of Bim levels were detected, and the combination of Mcl-1 and Bim changes appears to be closely correlated with the synergistic apoptosis (Fig. 1).

### Caspase-3 and caspase-8 are involved in the synergized cell death in RCC cells

**Fig. 2:**
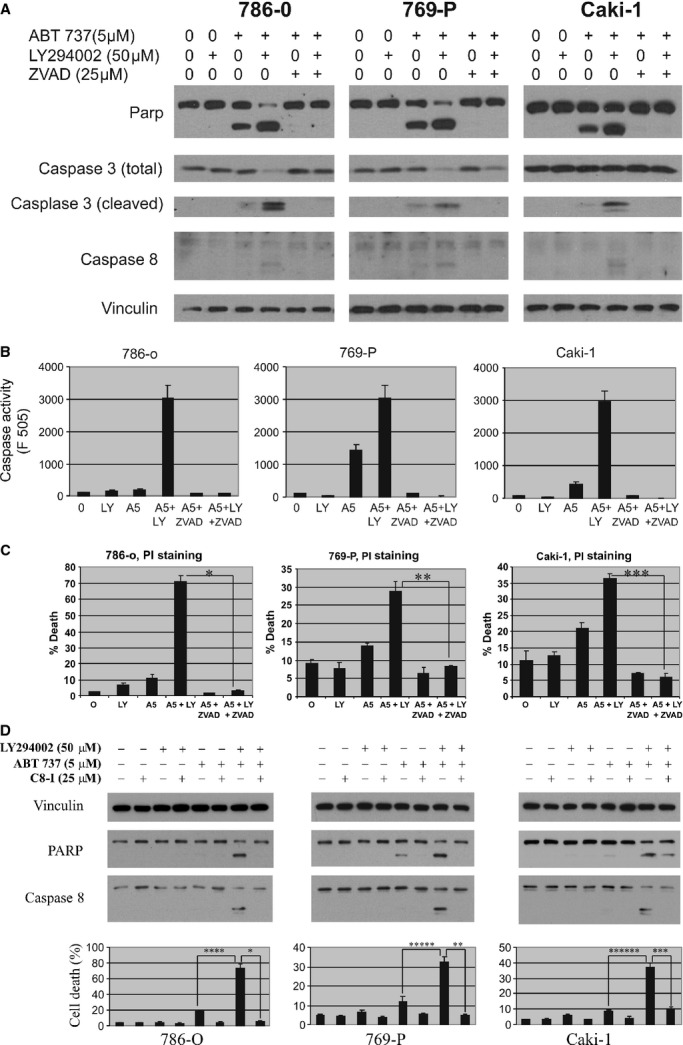
Caspase activation in RCC cells in treatment of LY 294002 and ABT-737. 786-O, 769-P and Caki-1 cells were first treated with either 50 μM LY 294002 in 0.1% DMSO or control 0.1% DMSO for 30 min. (with or without 25 μM ZVAD), followed by addition of 5 μM ABT-737 or control 0.1% DMSO for 3 hrs. Vinculin, PARP, caspase 3, caspase 3 cleaved and caspase 8 levels were assessed by immunoblotting with respective antibodies (**A**), and caspase activity was measured (**B**). Apoptosis was also assessed by flow cytometry with PI nuclear staining after 18 hrs incubation (**C**), *P < 0.001, **P < 0.005, ***P < 0.001, unpaired, one-tailed *t* test. (**D**) Effects of caspase 8 inhibitor on apoptosis and PARP cleavage were assessed as in (A), (B) & (C). *,**,***,****,*****,******P < 0.001, unpaired, one-tailed *t* test. The experiment was performed three times with similar results and representative blots were shown, *N* = 3.

To characterize the apoptotic pathways, we examined caspase activation in three RCC cell lines (786-O, 769-P, and Caki-1) treated with ABT-737 and LY 294002. Under conditions where LY 294002 (50 μM) and ABT-737 (5 μM) synergize to induce cell death, caspase 3 cleavage was significantly increased, with (786-O, 769-P) or without (Caki-1) apparent decrease in the levels of its full-length form (Fig. 2A), indicating activation of caspase 3. Also correlated with the synergistic cell death is the appearance of cleaved caspase 8, indicating activation of caspase-8 (Fig. 2A). The caspase activation was also confirmed by a fluorimetric assay (Fig. 2B). Furthermore, when the pan-caspase inhibitor ZVAD was added to the reaction, the enhanced cell death was completely blocked in all these cells (Fig. 2A–C). When specific caspase-8 inhibitor C8-I was added to the reaction, the enhanced cell death was also completely blocked in all these cells (Fig. 2D). These results suggested that the synergized cell death induced by LY 294002 and ABT-737 was mediated *via* caspase-dependant pathways with a particular dependence upon pathways mediated by Caspase 8.

### Cytochrome C level is enhanced in treatment of LY 294002 and ABT-737

**Fig. 3:**
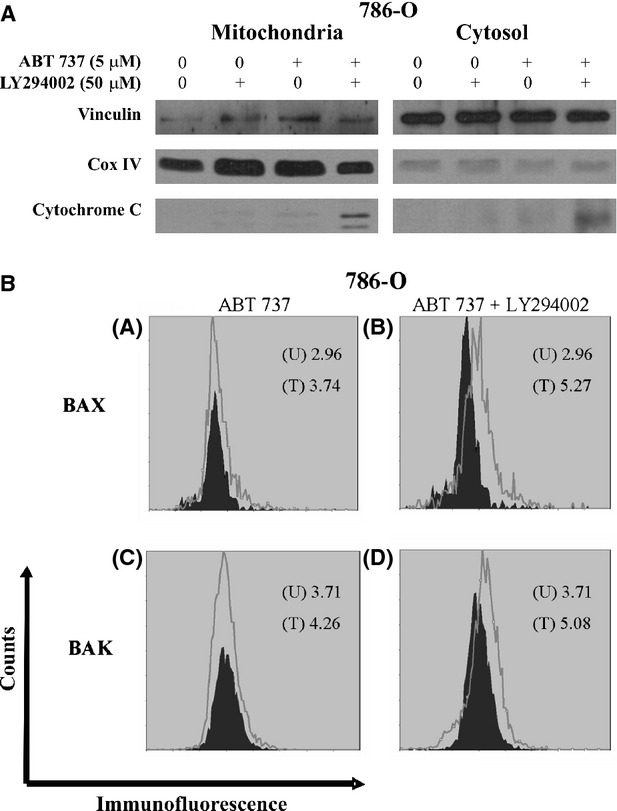
Effects of LY 294002 and ABT-737 on Cytochrome C levels and BAX and BAK activation in RCC. (**A**) 786-O cells were first treated with either 50 μM LY 294002 in 0.1% DMSO or control 0.1% DMSO for 30 min., followed by addition of 5 μM ABT-737 or control 0.1% DMSO for 3 hrs. Mitochondria were isolated from the cytosolic fraction in 786-O cells using the Mitochondria Isolation Kit for Mammalian Cells (Thermo Scientific). Vinculin, COX IV, and Cytochrome C levels were assessed by immunoblotting with respective antibodies. (**B**) Following treatment with 50 μM LY 294002 and 5 μM ABT-737 for 18 hrs, cells were probed with antibodies specific for the activated forms of BAX and BAK. BAX or BAK-related immunofluorescence was analysed by flow cytometry, and numbers representing the mean fluorescence intensities were shown. The experiments were performed three times with similar results and representative results were shown. *Filled histogram*: control untreated cells; *unfilled histogram*: treated cells.

Caspase activation is believed to be through either the mitochondria-mediated pathway or death receptor–mediated pathway. To determine if mitochondria is involved in the synergistic effect of these two drugs, we further examined the levels of cytochrome C in the cytosol. Vinculin and COX IV levels indicated that the separation of the mitochondria and cytosol was successful (Fig. 3A). While the levels of cytochrome C in the cytosol were increased slightly with LY 294002 and ABT-737 alone, its level was greatly increased by the combination of LY 294002 and ABT-737. This increase in cytosolic cytochrome C correlated with the increase in apoptosis and PARP cleavage in these cells (Figs 1 and 2), suggesting that the release of cytochrome C from mitochondria into the cytosol underlies the caspase activation and apoptosis. The increase in cytochrome C in the mitochondria during apoptosis has been reported previously [13].

### BAX and BAK levels are enhanced following treatment with LY 294002 and ABT-737

Next, we examined potential mechanisms which might explain the observed changes in mitochondrial membrane permeability. Bcl-xL and Bcl-2 were initially examined after treating 786-O cells with LY 294002 and ABT-737, and their levels remained unchanged as compared with non-treated controls (data not shown). We then measured the activation of BAX and BAK using flow cytometry utilizing antibodies that recognize the activated forms of the two proteins. As compared to the control untreated samples, treatment with ABT-737 alone increased the activation of BAX and BAK from 2.96 to 3.74 and 3.71 to 4.26, respectively, while treatment of the 786-O cells with both LY 294002 and ABT-737 increased the activation of BAX and BAK further to 5.27 and 5.08 (Fig. 3B), suggesting that the activation of BAX and BAK contributed to the change in the mitochondrial permeability that led to the release of cytochrome C in the cytosol.

### XIAP reduction is involved in LY 294002 and ABT-737 induced cell death

**Fig. 4:**
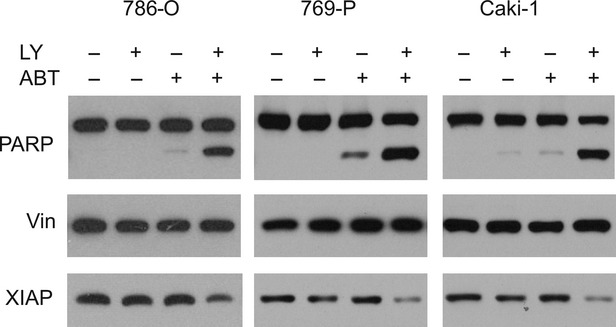
Levels of XIAP induced by LY 294002 and ABT-737 in RCC cells. Cells were first treated with either 50 μM LY 294002 in 0.1% DMSO or control 0.1% DMSO for 30 min., followed by addition of 5 μM ABT-737 or control 0.1% DMSO for 3 hrs. Vinculin, PARP and XIAP levels were assessed by immunoblotting with respective antibodies. The experiment was performed three times with similar results and representative blots were shown.

XIAP is a member of the inhibitor of apoptosis family of proteins (IAP), and XIAP inhibits apoptosis by directly binding and inhibiting caspases. We have screened various IAP family members, and found that while other IAP members remained invariant in abundance (data not shown) following treatment with LY 294002 and ABT-737, XIAP levels are significantly reduced in all cell lines. This significant reduction in XIAP level following treatment with LY 294002 and ABT-737 was correlated with the significant increase in PARP cleavage (Fig. 4), suggesting that XIAP may also contribute to the synergistic apoptosis induced by LY 294002 and ABT-737. Therefore, changes in XIAP, along with our previous observation of accompanying alterations in Bim and Mcl-1 (see Fig. 1), all likely contribute to the synergistic apoptosis induced by LY 294002 and ABT-737.

### XIAP, Mcl-1 and Bim collaborate in the synergistic apoptosis induced by LY 294002 and ABT-737

**Fig. 5:**
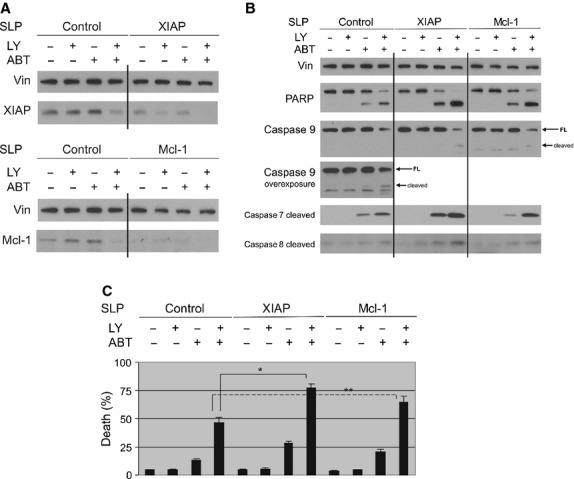
PARP cleavage and apoptosis induced by transduction with individual SLPs. 786-O cells were either transduced with control SLP or SLPs targeting the indicated genes of interest. Cells were then treated with either 50 μM LY 294002 in 0.1% DMSO or control 0.1% DMSO for 30 min., followed by addition of 5 μM ABT-737 or control 0.1% DMSO for 3 hrs. Vinculin, PARP, XIAP, Mcl-1, caspase 7, 8 and 9 levels were assessed by immunoblotting with respective antibodies and representative blots were shown. Apoptosis was also assessed by flow cytometry with PI nuclear staining after 18 hrs incubation *P < 0.001, **P < 0.01, unpaired, one-tailed *t* test. The experiment was performed three times with similar results and representative blots were shown.

As RCC cells are not transfected well, to examine if changes in XIAP, Mcl-1 and Bim levels could indeed affect apoptosis, we transduced 786-O cells with corresponding shRNA lentiviral particles (SLP), followed by examining changes in apoptosis and signalling. Unfortunately, Bim SLP failed to reduce Bim levels (data not shown), and was not used in any further experiments. The protein levels of XIAP and MCL-1 were reduced by the SLPs (Fig. 5A). Either Mcl-1 or XIAP knock down increased apoptosis (as measured by PARP cleavage), with XIAP having the greatest effect (Fig. 5B). This suggested that XIAP and Mcl-1 participate in the apoptosis in 786-O cells, and XIAP might be a more significant player than Mcl-1. This is further confirmed by the assays on caspases (Fig. 5B). While XIAP reduction appeared to cause more activation of caspase 7, 8 and 9 in the cells than did Mcl-1 reduction, reduction in XIAP and Mcl-1 both contributed to the caspase activation in the cells. The activation of caspase 7 and 9 fits the fact that they are direct targets of XIAP. Activation of caspase 9 in the presence of LY 294002 and ABT-737 (Fig. 5B) is also consistent with the fact release of cytochrome C from mitochondria to the cytosol is enhanced (Fig. 3A).

**Fig. 6:**
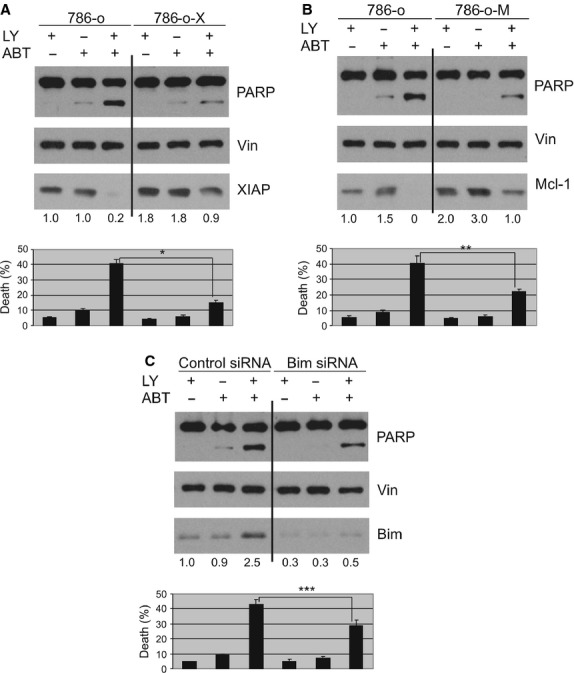
PARP cleavage and apoptosis induced by rescue of each gene expression. 786-O cells and stable cell lines (786-O-X, stable expression of XIAP; 786-O-M, stable expression of Mcl-1) were cultured in RPMI 1640 medium (**A** and **B**). 786-O cells were also transfected with control siRNA or siRNA targeting Bim for 24 hrs (**C**). Cells were then treated with either 50 μM LY 294002 in 0.1% DMSO or control 0.1% DMSO for 30 min., followed by addition of 5 μM ABT or control 0.1% DMSO for 3 hrs. Vinculin, PARP, XIAP, Mcl-1 and Bim levels were assessed by immunoblotting with respective antibodies. Apoptosis was also assessed by flow cytometry with PI nuclear staining after 18 hrs incubation *,**,***P < 0.01, unpaired, one-tailed *t* test. The experiment was performed three times with similar results and representative blots were shown.

We then examined the effect of knocking down Bim with siRNA, and overexpression of Mcl-1 and XIAP on the apoptosis. Reduction in Bim levels led to decreased PARP cleavage and apoptosis in the presence of LY 294002 and ABT-737 (Fig. 6C). On the other hand, overexpression of XIAP and Mcl-1 each led to decreased PARP cleavage and apoptosis in the presence of LY 294002 and ABT-737 (Fig. 6A and B). These results indicated that reduction in XIAP and Mcl-1, as well as the increase in Bim levels all contributed to the synergistic apoptosis induced by LY 294002 and ABT-737.

**Fig. 7:**
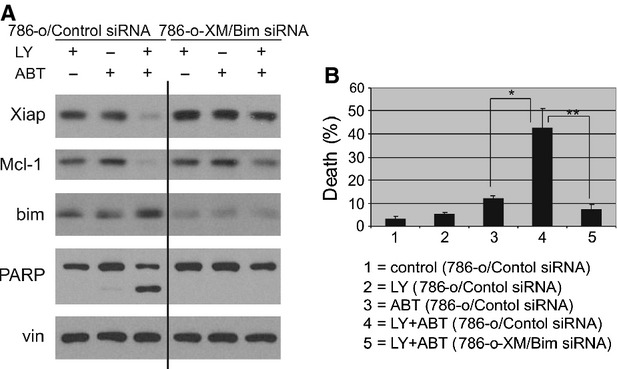
PARP cleavage and apoptosis induced by rescue of combined gene expression and knock down. 786-O cells were transfected with control siRNA and 786-O-XM cells stably expressing XIAP and Mcl-1were transfected with Bim siRNA. After 24 hrs, cells were then treated with either 50 μM LY 294002 in 0.1% DMSO or control 0.1% DMSO for 30 min., followed by addition of 5 μM ABT-737 or control 0.1% DMSO for 3 hrs. Vinculin, PARP, Mcl-1, Bim and XIAP levels were assessed by immunoblotting with respective antibodies. The experiment was performed three times with similar results and representative blots were shown (**A**). Apoptosis was also assessed by flow cytometry with PI nuclear staining after 18 hrs incubation, *N* = 3 (**B**). *P < 0.05, **P < 0.01, unpaired, one-tailed *t* test.

We then further examined the effect of knock down of Bim, along with simultaneous overexpression of both Mcl-1 and XIAP on the cells for treatment of LY 294002 and ABT-737. The combination of molecular rescue reverted the synergistic apoptosis induced by LY 294002 and ABT-737 almost completely, demonstrating that the synergistic apoptosis were through XIAP, Mcl-1 and Bim (Fig. 7).

## Discussion

While PI3-K inhibitors are being assessed clinically in RCC, it remains critical to identify mechanisms by which the therapeutic efficacy of these agents may be enhanced. In this manuscript, we report a strategy where simultaneous targeting of the PI3-K and Bcl-2 family can significantly increase the cell death in RCC cells. Results obtained from exploring the mechanism behind this enhanced effect on cell death also provide an understanding of the relative resistance of RCC cell lines to PI3-K inhibitors alone.

Synergy or additive effects between PI3K/AKT pathway and Bcl-xL in controlling apoptosis have been noted in lung cancer [14]. However, in contrast to lung adenocarcinoma cells in which Bim appears to play the major role in the synergized apoptosis, XIAP, Mcl-1 and Bim all contributed to the synergized apoptosis in RCC cells. Results of Figure 6 suggested that among the three molecules, XIAP and to a lesser extent, Mcl-1, appear to be the more important players in the synergized apoptosis observed in RCC cells.

We hereby first described XIAP reduction in response to LY 294002 and ABT-737. XIAP is possibly the only mammalian IAP that inhibits caspase activity directly [15]. Enhanced XIAP expression has been suggested to be responsible for drug resistance of RCC [16, 17]. Our SLP and overexpression results further confirmed the significance of XIAP in the RCC drug resistance; by using LY 294002 and ABT-737, we have also found a way to reduce XIAP levels in RCC, which may be a clinically feasible way of targeting a protein associated with RCC drug resistance.

The fact that Mcl-1 is involved in the synergized apoptosis is also interesting. It has been reported that PI3K upregulates Mcl-1 [18, 19]. However, we have not seen significant downregulation of Mcl-1 when PI3K was inhibited alone. Instead, the loss of Mcl-1 was only observed when LY 294002 and ABT-737 were simultaneously used. It's also interesting to note that although ABT 737 is an antagonist of Bcl-2, Bcl-xL and Bcl-w, it is not of Mcl-1; yet in combination with PI3K inhibitor, Mcl-1 was successfully reduced. The fact that Mcl-1 has been reported to be the primary protein responsible for the resistance to ABT 737 in small cell lung cancer and myeloid leukaemia [20, 21] has also suggested that reduction in Mcl-1 would be an important cancer therapeutic target.

In summary of the plausible mechanism underlying the synergistic cell death induced by ABT 737 and LY 294002, reduction in Mcl-1 promotes the release of the apoptotic binding partner Bim from Mcl-1 complexes; this further increased the effect of increased level of Bim, which through binding BAX (or BAK) [22], activates BAX, and leads to the release of cytochrome C [23], followed by activation of Apaf-1 and Caspase 9, and effector caspases thereafter. Furthermore, the reduction in XIAP reduces its ability to bind and inhibit caspase 3, 7 and 9, leading to a more significant level of apoptosis.

Caspase 8 activation has been best known to be typically induced by extrinsic pathways, *i.e*. ligand-induced activation of death receptors followed by recruitment and activation of Fas-associated death domain (FADD), which then recruits caspase-8. As caspase 8 was involved in the synergistic apoptosis we observed, we have also examined the possibility that extrinsic pathway is involved in the induced apoptosis. However, we have not detected activation or elevation of the death receptors (data not shown). As it is known that initiator caspase 8 is also part a of the caspase 3, 6, 8 loop and is subject to the regulation by effector caspase 3, 6 [24], it is plausible that caspase 8 activation and the observed apoptosis is completely intrinsic. Caspase 3 activation may lead to caspase 8 activation, followed by amplified caspase 3, 6 activation and enhanced apoptosis.

Although we have demonstrated the roles of XIAP, Mcl-1, and Bim in the apoptosis induced by LY 294002 and ABT-737, the mechanism by which these two drugs act together to regulate XIAP, Mcl-1 and Bim remains unknown. Further studies will shed light on details of their regulation. The variation in the extent of synergy or additive effects on apoptosis among different RCCs is probably due to difference in cellular contexts among different RCC cells. For example, the AKT activity (phospho-AKT) is lower in 769-P cells than in 786-O cells, thus 769-P cells have a higher basal level of cell death and are more sensitive to individual apoptosis stimuli. Regardless, out results suggest that the strategy of combining PI3-K inhibitors with BH3-mimetics in RCC may be viable therapeutic approach.
