# Corrigendum

**DOI:** 10.1111/jcmm.12323

**Published:** 2014-07-30

In [1], the author affiliations, and corresponding author information were incorrect in some parts. Please find the correct author affiliations and corresponding author information below.

Author affiliations:

Shudong Zhu^a, b,^ *, Matthew B. Cohen^b^, Jeffrey D. Bjorge^c^, James W. Mier^b^, Daniel C. Cho^b,^ *

^a^School of Life Sciences, State Key Laboratory of Medical Genetics, Central South University, Changsha, China

^b^Division of Hematology and Oncology, Beth Israel Deaconess Medical Center, Harvard Medical School, Boston, MA, USA

^c^Department of Biochemistry and Molecular Biology, Southern Alberta Cancer Research Institute, Calgary, Canada

Corresponding author information:

*Correspondence to: Shudong ZHU, Ph.D., School of Life Sciences, Central South University, 172 TongZiPo Rd, Changsha, China.

Tel.: 731-8265-0460

Fax: 731-8265-0460

E-mail: shudongzhu@csu.edu.cn

The authors wish to apologize for any misunderstanding or inconvenience caused.
